# Microbial Community and Short-Chain Fatty Acid Mapping in the Intestinal Tract of Quail

**DOI:** 10.3390/ani10061006

**Published:** 2020-06-09

**Authors:** Xizhong Du, Yun Xiang, Fangfang Lou, Pingguang Tu, Xiaojun Zhang, Xujin Hu, Wentao Lyu, Yingping Xiao

**Affiliations:** 1 Institute of Animal Husbandry and Veterinary Medicine, Jinhua Academy of Agricultural Sciences, Jinhua 321011, China; duxizhong@jhnky.cn (X.D.); xiangyun@jhnky.cn (Y.X.); loufangfang@jhnky.cn (F.L.); tupingguang@jhnky.cn (P.T.); zhangxiaojun@jhnky.cn (X.Z.); huxujin@jhnky.cn (X.H.); 2 State Key Laboratory for Managing Biotic and Chemical Threats to the Quality and Safety of Agro-products, Institute of Quality and Standard for Agro-products, Zhejiang Academy of Agricultural Sciences, Hangzhou 310021, China; lvwt@zaas.ac.cn

**Keywords:** quail, intestine, microbiota, short-chain fatty acids

## Abstract

**Simple Summary:**

Quail is an economically important type of poultry, valued for its high meat quality and abundant egg nutrition. It is also an important laboratory research animal, widely used in developmental biology and toxicology tests. Since the gut microbiota plays a vital role in the host’s growth and health, we investigated the microbiota inhabiting the duodenum, jejunum, ileum, cecum, and colorectum of quail in the present study, using 16S rRNA gene sequencing and qPCR. The concentrations of short-chain fatty acids (SCFAs) were evaluated using gas chromatography. We found that the microbiota in the cecum was different from other intestinal sections and the enriched inhabitants of SCFA-producing bacterial genera made cecum the core locations of SCFA production in quail. The results of this study will provide fundamental data for further quail microbiology and functional studies.

**Abstract:**

Quail is raised throughout China for egg and meat production. To deeply understand the gastrointestinal microbial composition and metabolites of quail, the present study characterized the microbiota inhabiting five intestinal locations of eight-week-old quail using 16S rRNA gene sequencing and qPCR, and evaluated the concentrations of short-chain fatty acids (SCFAs) in each individual location using gas chromatography. The results showed that Firmicutes, Bacteroidetes, Proteobacteria, Actinobacteria, and Deferribacteres were the five most abundant phyla in the intestinal tract of quail. Firmicutes was largely dominant (>95%) in the small intestine, whereas Bacteroidetes increased significantly in the cecum (19.19%) and colorectum (8.09%). At the genus level, *Lactobacillus* was predominant in almost all sections (>50%) except in the cecum (7.26%), where *Megamonas*, *Faecalibacterium*, and *Bacteroides* were dominant. qPCR data indicated that the population sizes of both the total bacteria and proportions of the Firmicutes, Bacteroidetes, and *Bacteroides* group increased going from the proximal toward the distal end of the intestine in quail. The SCFA-producing bacterial genera *Bacteroides*, *Faecalibacterium*, *Alistipes*, *Blautia*, *Parabacteroides*, and *Clostridium* were of higher richness in the cecum and colorectum, where, accordingly, more SCFAs were produced. These findings will be helpful for the future study of quail microbiology, as well as its relationship with productive performance and health.

## 1. Introduction

Quail (*Coturnix*) is an economically important poultry, for its high meat quality and profuse egg nutrition. Moreover, its early sexual maturity, high laying rate, short generation interval, rapid growth, and the limited feed and space required lead to fast returns on investment [1,2], which have made quail breeding develop into the third largest poultry industry after chicken and duck in some Asian countries. Additionally, quail is an important laboratory research animal, which is widely used in developmental biology and toxicology tests [3,4,5].

The quail gastrointestinal tract (GIT) is inhabited by various microbial populations [6]. The gastrointestinal microbiota play an important role in the host’s nutritional, physiological, and immunological processes [7,8,9], by contributing to the enhancement of nutrient absorption, the development of the immune system, and prevention of colonization by pathogens [6,10]. A mutualistic relationship between the gastrointestinal microbiota and the host is critical for poultry [11]. One notable example of such a relationship is the generation of short-chain fatty acids (SCFAs) through bacterial fermentation of nondigestible carbohydrates [12]. SCFAs produced by bacteria provide an energy source to the host and mitigate inflammation, as well as regulate, among other things, satiety [13]. Aetate, propionate, and butyrate are the main categories of SCFAs in animals, including humans [12,14].

China is currently the largest quail breeding country, accounting for around one-fifth of quail in the world. The quail line originating from Japanese quail (*Coturnix japonica*) was introduced into China from Korea in the 1970s and has been widely bred, due to its fast growth and high laying rate. At present, this quail line is the main breed of egg quail in China and is widely used to establish synthetic lines of egg quail. Although a few recent studies have provided insights into the gastrointestinal microbiota of the northern bobwhite quail [15] and Japanese quail [6], based on the analysis of cultivable bacteria and 16S rRNA gene sequencing, respectively, knowledge about the spatial variation of gut microbial community in quail remains limited. In this study, we present the profiles of the microbial community, as determined using high-throughput sequencing and further identify some key microbial populations, by qPCR, in different locations of the quail intestinal tract. Furthermore, the SCFAs in different intestinal sections were also evaluated by gas chromatography (GC). Since the duodenum, jejunum, ileum, cecum, and colorectum are the main gut regions where microbiota contribute to the poultry productivity and disease resistance [16], the microbial diversity and community structure were investigated from these five different intestinal sections of quail.

## 2. Materials and Methods

### 2.1. Ethical Statement

The animal experimental procedures were approved by the Institutional Animal Care and Use Committee of Jinhua Academy of Agricultural Sciences (JHNKY2018-006).

### 2.2. Animals, Experimental Design and Sample Collection

A flock of 200 day-of-hatch female quail were obtained from a commercial hatchery (Jinhua Hurui Breeding Co. Ltd, Jinhua, China), which was of the line originating from Japanese quail, and introduced into China from Korea. The quail were housed together in a rearing pen under standard management, at 34–36 °C for the first three days and at 32–34 °C for 4–7 d, followed by a reduction by 2–3 °C per week to a final temperature of 26 °C. At 3 weeks of age, birds were housed at 10 per cage. Feed (Table 1) and water were provided ad libitum. The starter feed was replaced with grower feed at 4 weeks of age. At 8 weeks of age, all the birds were weighed to obtain average body weight, and 8 birds with an average body weight (144.05 ± 5.44 g) were then selected from different cages and euthanized by cervical dislocation. The intestine was immediately removed from the carcasses, and the luminal contents from the duodenum, jejunum, ileum, cecum, and colorectum were collected. All samples were frozen immediately in liquid nitrogen and stored at −80 °C until further analysis.

### 2.3. DNA Extraction, Amplification and Sequencing

Genomic DNA from the contents of each section of quail intestinal tract was extracted using the QIAamp DNA Stool Mini Kit (QIAGEN, Valencia, CA, USA), according to the manufacturer’s instructions.

Amplicons from the V3–V4 region of bacterial 16S rRNA genes were produced using the barcode-fusion primers pairs: 338F (5′-CTACGGGNGGCWGCAG-3′) and 806R (5′-GACTACHVGGGTWTCTAAT-3′) and then sequenced on the Illumina MiSeq 2 × 300 platform. Raw reads were assigned to each sample according to the unique barcode and overlapped using FLASH. To obtain clean sequencing data, the chimeric reads were identified and removed by using USEARCH, following the UCHIME algorithm [17]. The analyses of gut microbial diversity and taxonomy assignment of OTUs were performed by QIIME software with default parameters [18]. The representative OTU sequences were annotated with taxonomic information of the SILVA database using the RDP classifier [19]. The Venn diagram was generated using the R package (http://www.R-project.org/). Alpha diversity (Observed_species, Chao1, Shannon and Simpson indices), β diversity (weighted UniFrac distance), as well as the relative abundance of bacteria at phylum and genus levels, were analyzed on the normalized OTU table with the scripts alpha_diversity.py, beta_diversity_through_plots.py, and summarize_taxa_through_plots.py, respectively. Goods_coverage was used to determine their representation in a sample as a proportion of total species. The rarefaction curves that characterized sequencing depth were generated based on Observed_species. An analysis of similarities (ANOSIM) was used to detect statistical differences of UniFrac distance metric with the script of compare_categories.py. Principal component analysis (PCA) was conducted to illustrate the β-diversity based on weighted UniFrac distances.

### 2.4. Analysis of Short-Chain Fatty Acids

Concentrations of SCFAs in intestinal contents were detected with the gas chromatographic method, as described in our previous report [20]. Briefly, 100 mg of the luminal content of each intestinal section was vigorously vortex-mixed with ten times deionized water. After the mixture was centrifuged (12,000 rpm for 10 min), 500 μL aliquots of the supernatant were added to 100 μL of a 25% (*w*/*v*) crotonic acid (internal standard) solution in metaphosphoric acid. The mixed solution was filtered with a 0.22 μm mesh and was then used to measure the contents of SCFAs by gas chromatography (GC-2010 plus, Shimadzu, Kyoto, Japan).

### 2.5. qPCR Analyses of Key Bacteria and Genes in Butyrate Production

The gene copy number of the terminal genes for butyrate synthesis, butyrate kinase and butyryl CoA:acetate CoA transferase corresponding to total bacteria, Firmicutes and Bacteroidetes phyla, *Bacteroides* genus, was assessed in quail intestinal contents by qPCR on an ABI Prism 7700 Sequence Detector (Applied Biosystems, Foster City, CA, USA), using the extracted DNA as templates and SYBR Green PCR Master Mix (Takara, Tokyo, Japan), as previously described [21,22]. DNA was amplified under the following conditions: 95 °C for 2 min, followed by 35 cycles of 15 s at 95 °C, 45 s at 58 °C, and 1 min at 72 °C. Each sample was analyzed in triplicate. The used primer sets are listed in Table 2. A melting curve analysis was performed after each amplification to confirm specificity of the reaction. Quantification was done by using standard curves made from known concentrations of plasmid DNA, containing the respective amplicon for each set of primers. All qPCR results were expressed as gene copies per gram of luminal contents.

### 2.6. Statistics

Statistical analyses and graphing were conducted using SPSS statistics software (version 20.0, International Business Machines Corporation, Armonk, NY, USA) and GraphPad Prism (version 6.0, GraphPad Software Inc., San Diego, CA, USA). Data are expressed as means ± SEM. Statistical comparisons of the microbial composition between the different intestinal sections were performed using an ANOVA analysis, accompanied with Tukey’s honestly significant difference post-hoc test. Test results of all analyses were considered significant when *p* < 0.05. The correlation between bacterial genera and SCFAs was estimated by Spearman correlation analysis in R (version 3.6.3, https://www.r-project.org/).

## 3. Results

### 3.1. Microbial Complexity

**Figure 1:**
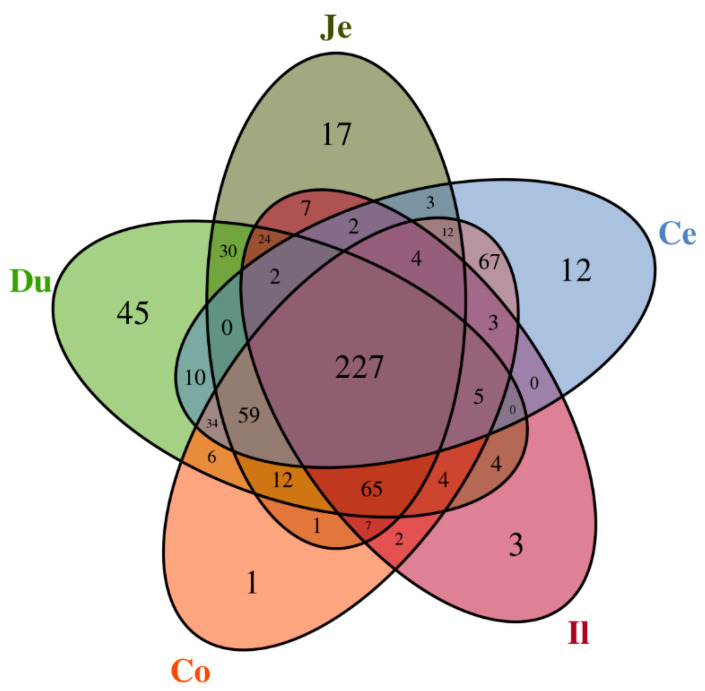
OTUs shared or unshared by the five intestinal sections of quail. The Venn diagram shows the numbers of OTUs (97% sequence identity), shared or unshared by the duodenum (Du), jejunum (Je), ileum (Il), cecum (Ce), and colorectum (Co), respectively, depending on the overlaps.

A total of 2,413,643 sequences with an average length of 427 bp were obtained from 40 samples, with the sequence number ranging from 55,187 to 64,608 per individual after quality filtering, and clustered into 668 OTUs (ranging from 141 to 353 for each sample), at the 97% sequence similarity value. As shown in the Venn diagram (Figure 1), 227 OTUs were shared by the five intestinal sections, and unique OTUs that only presented in one section varied from 1 (colon) to 45 (duodenum). The other OTUs were shared by 2–4 intestinal sections.

Microbial complexity in the duodenum, jejunum, ileum, cecum, and colon was estimated by calculating the alpha diversity indices (Table 3). Shannon and Simpson indices were used to describe the bacterial community diversity, which were higher in cecum and colon samples than in small intestinal sections. These data suggested that the microbiota in the large intestine were of higher richness and more diverse than in the small intestine, where the duodenum exhibited a higher community richness and diversity compared to the jejunum and ileum. The Good’s coverage estimators of the samples ranged from 0.9989 to 0.9992, suggesting that there was a very low probability that there was an amplicon not being sequenced in the sample. Therefore, the sequencing results are considered to adequately represent the bacterial diversity of the entire sampled population.

### 3.2. Microbial Community Composition across the Intestinal Tract

**Figure 2:**
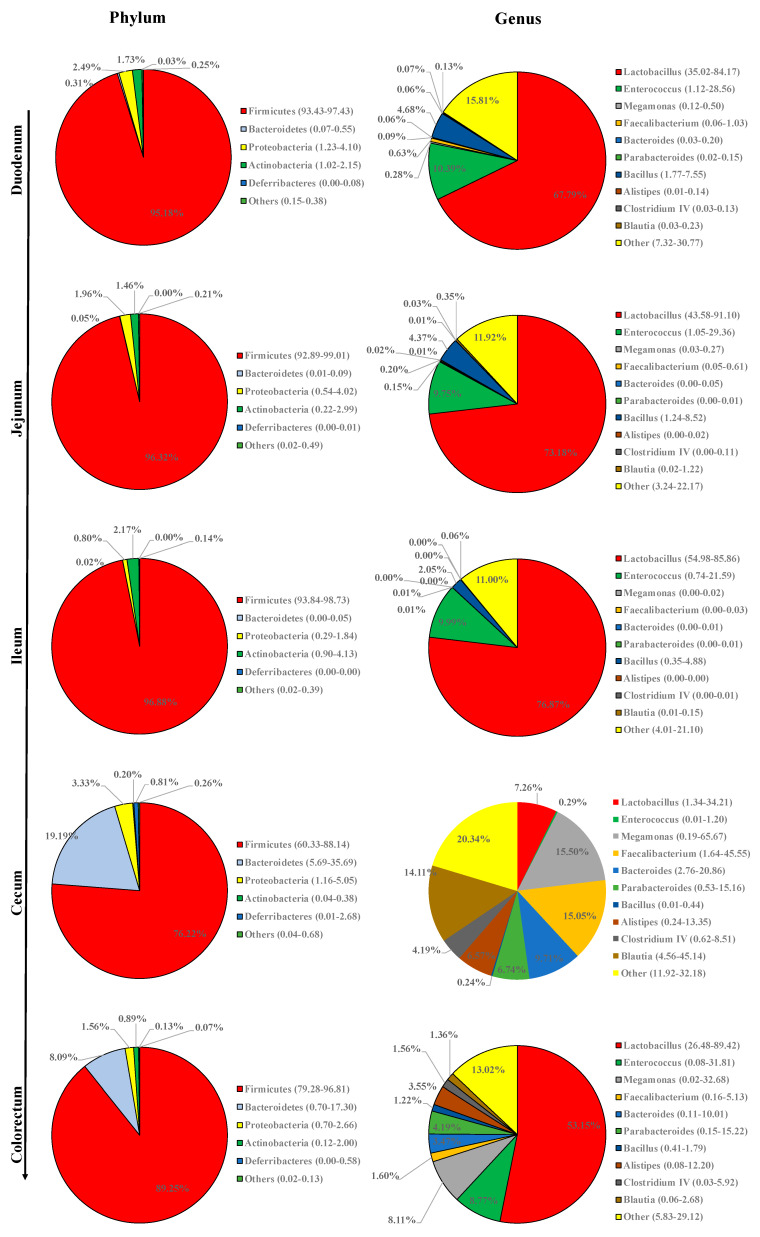
The composition of gut microbiota in the five intestinal sections of quail. The top five phyla and top 10 genera are shown.

**Figure 3:**
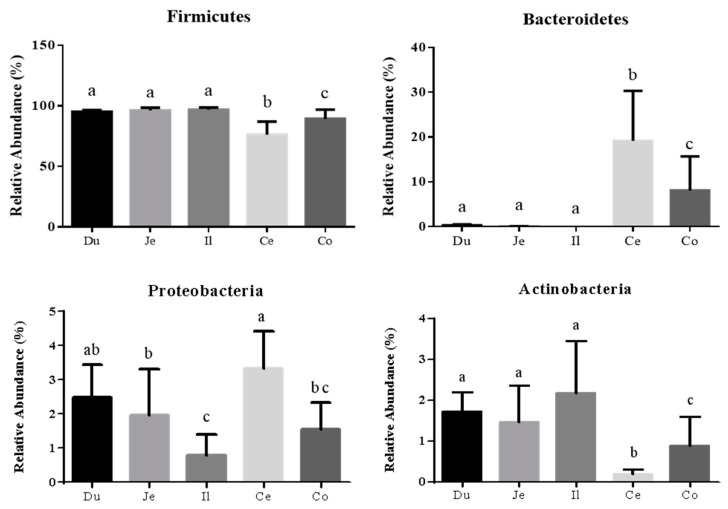
Differences in the four most abundant phyla among the five intestinal sections of quail. Different letters indicate significant difference. The error bars represent standard deviations. Du: duodenum; Je: jejunum; Il: ileum; Ce: cecum; Co: colorectum.

The overall intestinal tract of quail was inhabited by more than 15 phyla identified by sequence clustering. The five most abundant phyla were Firmicutes, Bacteroidetes, Proteobacteria, Actinobacteria, and Deferribacteres (Figure 2). As shown in Figure 3, Firmicutes was largely dominant in all sections and accounted for >95% of the total sequences in the duodenum, jejunum, and ileum. The proportion of Bacteroidetes increased dramatically in cecum and colorectum, with the proportions of Firmicutes and Actinobacteria correspondingly showing a significant decrease.

**Figure 4:**
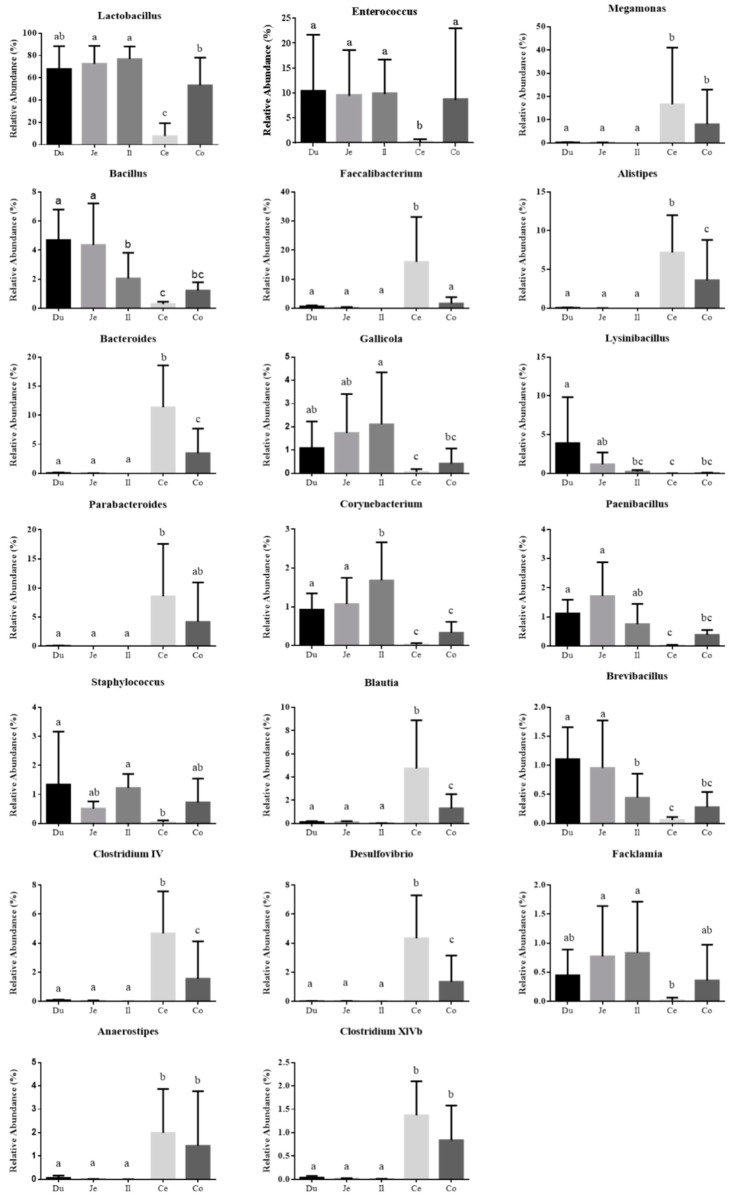
Differences in the 20 most abundant genera among the 5 intestinal sections of quail. Different letters indicate significant difference. The error bars represent standard deviations. Du: duodenum; Je: jejunum; Il: ileum; Ce: cecum; Co: colorectum.

The 20 most abundant genera are presented in Figure 4, including Lactobacillus, Enterococcus, Megamonas, Bacillus, Faecalibacterium, Alistipes, Bacteroides, Gallicola, Lysinibacillus, Parabacteroides, Corynebacterium, Paenibacillus, Staphylococcus, Blautia, Brevibacillus, Clostridium IV, Desulfovibrio, Facklamia, Anaerostipes, and Clostridium XIVb. In most samples, the top 10 genera accounted for nearly or more than 80% of the total sequences (Figure 2). Lactobacillus was the predominant genus in the duodenum, jejunum, ileum, and colorectum, followed by Enterococcus. The bacterial community structure was apparently different in the cecum, as characterized by the remarkably reduced Lactobacillus and relatively even distribution of other genera. Enterococcus, Staphylococcus, and Facklamia were also decreased in the cecum, while Faecalibacterium was notably increased. Moreover, compared with those in the small intestine (the duodenum, jejunum, and ileum), Megamonas, Alistipes, Bacteroides, Parabacteroides, Blautia, Clostridium IV, Desulfovibrio, Anaerostipes, and Clostridium XIVb were more enriched, while Bacillus, Gallicola, Corynebacterium, Paenibacillus, and Brevibacillus were considerably less abundant in the cecum and colorectum. The representation of Lysinibacillus diminished gradually along the intestine tract.

To further confirm the bacterial population in different intestinal tract sections of quail, the gene copy number selected genes corresponding to total bacteria, Firmicutes and Bacteroidetes phyla, and the genus *Bacteroides* were examined by qPCR. As listed in Table 4, a significant increase in total bacteria and the representatives of the Firmicutes and Bacteroidetes, the latter of which includes the genus *Bacteroides*, was observed, going from the proximal to distal intestinal tract.

### 3.3. Similarity of Microbiota across the Intestinal Tract

**Figure 5:**
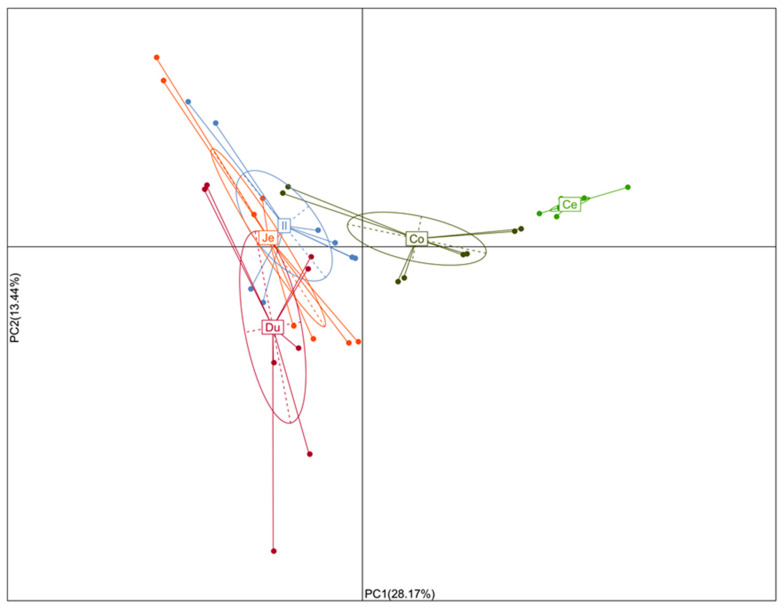
Principal component analysis (PCA) of the dissimilarity of microbial community compositions among the samples based on weighted Unifrac distance. Du: duodenum; Je: jejunum; Il: ileum; Ce: cecum; Co: colorectum.

To evaluate the similarity and difference of the bacterial community structures in the five intestine sections from eight quail, a principal component analysis (PCA) based on weighted UniFrac distance metrics was performed. PC1 and PC2 accounted for 28.17% and 13.44% of the total variation, respectively. As delineated in Figure 5, the microbiota of small intestine sections (the duodenum, jejunum, and ileum), the cecum, and colorectum were significantly different from one another by PC1, and samples of the duodenum, jejunum, and ileum were further separated by PC2.

### 3.4. SCFAs in Different Intestinal Tract Sections and Its Correlation with Microbiota

The SCFA concentrations varied in different anatomical regions in the quail intestine (Table 5). Acetate and propionate were present in all sections; butyrate was found in the ileum, cecum, and colorectum; isobutyrate and valerate were found in the cecum and colorectum, while isovalerate were found only in the cecum. The concentrations of total SCFAs in the cecum were significantly higher than those in other sections (*p* < 0.05).

**Figure 6:**
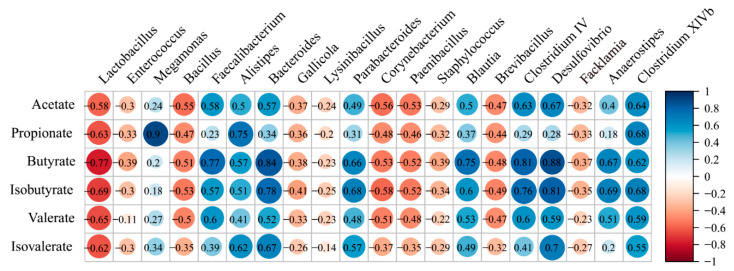
Correlation coefficient between the top 20 genera and SCFAs in intestinal tract.

The SCFA concentrations were strongly correlated with the relative abundance of Faecalibacterium, Alistipes, Bacteroides, Parabacteroides, Blautia, Clostridium IV, Desulfovibrio, Anaerostipes, and Clostridium XIVb (Figure 6).

### 3.5. Genes for Butyrate Synthesis in Different Intestinal Tract Sections

The terminal genes for butyrate synthesis, butyrate kinase, and butyryl CoA:acetate CoA transferase in luminal contents of each intestinal sections were further assessed in terms of copy number using qPCR. As shown in Table 6, significantly more copies of butyrate kinase and butyryl CoA:acetate CoA transferase genes were detected in cecum samples, consistent with the highest level of butyrate in the cecum.

## 4. Discussion

The quail line introduced from Korea is widely raised throughout China for egg production. The unique microbial metabolism in the gastrointestinal tract allows the bird to efficiently digest its food with a much shorter transit time than for mammals [6,23]. Numerous investigations have indicated that the gut microbiota plays a vital role in nutrient absorption, immune response, physiological metabolism, and organ development of the host [24,25]. Disturbance of gut microbiota in poultry can result in increased susceptibility to pathogen colonization and cause serious losses to farmers [26,27,28]. Therefore, the characterization of the gastrointestinal microbial community in healthy quail may help to understand and predict changes in microbiota related to physiological and pathological states.

The quail intestinal tract is composed of the duodenum, jejunum, ileum, cecum, and colorectum, which are distinguished from each other in function and physiochemical characteristics, as well as being inhabited by a specialized microbial community [6,15]. The present study represents the first report on the spatial patterning of the microbial community in the intestinal tract of healthy quail based on 16S rRNA gene sequencing. Alpha diversity indices were used to describe the bacterial community richness (Observed species and Chao1 estimator) and diversity (Shannon and Simpson indices), all of which descended along the small intestine sections and then ascended significantly in the cecum and colorectum. That is, the richness and diversity of microbiota was highest in the cecum and lowest in the ileum, which was consistent with the results found in other poultry, such as chickens [23,29], ducks [30], and geese [31]. Beta diversity was illustrated by principal component analysis (PCA) based on weighted UniFrac distances, and showed a clear separation of the cecum, colorectum, and small intestine sections.

The microbial community composition analysis showed that Firmicutes, Bacteroidetes, Proteobacteria, and Actinobacteria were the dominant phyla with the highest abundances in the intestine of quail, which have also been determined to be predominant in the GIT of other birds [29,31,32]. As for the spatial distribution, distinct microorganisms prefer different gastrointestinal sections [29,31,33]. For example, Bacteroidetes was found to be considerably more abundant in the cecum than in the other intestinal sections of quail, with similar trends observed in previous reports on Japanese quail [6], turkeys [34], chickens [29] and geese [31]. At the genus level, *Lactobacillus* was dominant in the duodenum, jejunum, ileum, and colorectum, while being remarkably reduced in the cecum, where *Faecalibacterium*, *Bacteroides*, *Alistipes*, *Parabacteroides*, and *Anaerostipes* were notably increased, consistent with the genera distribution found in Japanese quail [6] and our previous findings on broiler chickens [29] and geese [31]. The greater diversity of bacterial genera in the cecum may contribute to the function of the avian cecum as a vault that protects microbial diversity and re-seeds the GIT after significant disturbance or acute dysbiosis [33,35]. Additionally, the population of total bacteria and the representatives of Firmicutes, Bacteroidetes, and *Bacteroides* as measured by qPCR were higher in the cecum than in the small intestine of GIT in quail, probably owing to the gradually increased luminal pH and sharply decreased oxygen concentrations going from the proximal to the distal GIT, which results in the bulk of bacterial growth being present in the large intestine of other animals, as has been reported [36,37].

SCFAs are intestinal fermentation products that contribute to energy homeostasis and the physiological processes of the host [9,12,37]. Here, we found that the cecum generated the most SCFAs in the quail intestine, followed by the colorectum, which was positively correlated to the richness of the SCFA-producing bacterial genera *Bacteroides*, *Faecalibacterium*, *Alistipes*, *Blautia*, *Parabacteroides*, *Anaerostipes*, and *Clostridium* [14,38,39]. Additionally, *Faecalibacterium*, *Alistipes*, *Blautia*, *Anaerostipes*, and *Clostridium IV* have a high capacity for producing butyrate through the two main pathways, the butyryl CoA:acetate CoA transferase pathway and the butyrate kinase pathway [21,40,41]. We further assessed these two genes of butyrate synthesis pathways by qPCR, and detected more gene copies of butyrate kinase and the butyryl CoA:acetate CoA transferase in the cecum and colorectum samples, in line with the higher concentrations of butyrate in the two sections. This illustrated that SCFAs are more abundant in the cecum, compared to the other GIT sections, due to the increased richness of bacteria harboring SCFA-producing genes and, therefore, higher levels of produced SCFAs. However, the quail in the present study were from only one hatchery. In future, systematic studies of the gut microbiota in quail of different ages and from different hatcheries are need to determine the potential effect of these factors on metabolite and production.

## 5. Conclusions

This work presents the first overview of the microbial community and SCFA concentrations in five intestinal locations of quail. It showed that Firmicutes, Bacteroidetes, Proteobacteria, Actinobacteria, and Deferribacteres were the five most abundant phyla in the intestinal tract of quail. Firmicutes was largely dominant in all sections, though the representation of Bacteroidetes increased dramatically in the cecum and colorectum. *Lactobacillus* was predominant in almost all sections of quail intestine except in the cecum, where *Megamonas*, *Faecalibacterium*, and *Bacteroides* were dominant. The population of the total bacteria and the representatives of the Firmicutes, Bacteroidetes, and *Bacteroides* were increased going from the proximal to distal intestine in quail. The enrichment of SCFA-producing bacterial genera *Bacteroides*, *Faecalibacterium*, *Alistipes*, *Blautia*, *Parabacteroides*, *Anaerostipes* and *Clostridium* made the cecum and colorectum the core locations of fiber fermentation and SCFA production in quail. Collectively, these findings improve our understanding of the intestinal microbiota of quail and may provide information on the relationship between intestinal microbiota and the host’s productive performance and health.

## Figures and Tables

**Table 1:** Composition and nutrient levels of diets.

Item	Starter Feed	Grower Feed
Ingredient (% of diet)		
Corn	53.24	58.88
Soybean meal	38.66	32.97
Soybean oil	2.80	3.29
CaHPO_4_	1.26	1.09
Limestone	1.31	1.41
NaCl	1.26	1.09
Met	0.24	0.22
Lys	0.03	0.05
Premix	1.00	1.00
Nutrient level		
Metabolizable energy, MJ/kg	12.13	12.65
Crude protein, %	20.80	19.50
Lys	1.28	1.19
Met	0.52	0.45
Calcium, %	0.91	0.85
Phosphorus, %	0.57	0.53

The premix provided per kilogram of total diet: vitamin A: 10,000 IU; vitamin D3: 2100 IU; vitamin E: 15 IU; vitamin K3: 1 mg; vitamin B1: 2 mg; vitamin B2: 4 mg; vitamin B6: 3 mg; vitamin B12: 0.005 mg; nicotinic acid: 40 mg; pantothenic acid: 10 mg; folic acid: l mg; biotin: 0.3 mg; choline: 2000 mg; Fe: 120 mg; Cu: 5 mg; Mn: 60 mg; Zn: 25 g; I: 0.3 mg; Se: 0.2 mg.

**Table 2:** Primers used in the present study.

Item	Primers (5′–3′)	Reference
Total Bacteria	fwd CGGYCCAGACTCCTACGGG rev TTACCGCGGCTGCTGGCA	[21]
Firmicutes	fwd GGAGYATGTGGTTTAATTCGAAGCA rev AGCTGACGACAACCATGCAC	[21]
Bacteroidetes	fwd GGARCATGTGGTTTAATTCGATGAT rev AGCTGACGACAACCATGCAG	[21]
Bacteroides	fwd GAGAGGAAGGTCCCCCAC rev CGCTACTTGGCTGGTTCAG	[22]
Butyryl-CoA:acetate-CoA transferase	fwd AAGGATCTCGGIRTICAYWSIGARATG rev GAGGTCGTCICKRAAITYIGGRTGNGC	[21]
Butyrate kinase	fwd TGCTGTWGTTGGWAGAGGYGGA rev GCAACIGCYTTTTGATTTAATGCATGG	[21]

**Table 3:** Overview of sequencing results and alpha diversity indices of samples.

Item	Duodenum	Jejunum	Ileum	Cecum	Colorectum
Sequencing Number	60280 ± 3003	60591 ± 2437	61195 ± 2438	60101 ± 3778	59540 ± 1336
OTUs	259 ± 23	237 ± 68	201 ± 40	275 ± 39	270 ± 45
Chao1	281.67 ± 29.79 ^ab^	271.10 ± 75.05 ^ab^	257.66 ± 29.92 ^b^	310.72 ± 51.10 ^a^	322.06 ± 61.66 ^a^
Shannon	3.07 ± 0.45 ^bc^	2.78 ± 0.77 ^c^	2.61 ± 0.47 ^c^	4.83 ± 0.52 ^a^	3.72 ± 0.76 ^ab^
Simpson	0.71 ± 0.08 ^b^	0.68 ± 0.10 ^b^	0.68 ± 0.07 ^b^	0.89 ± 0.06 ^a^	0.81 ± 0.08 ^ab^
Good’s coverage	0.9992 ± 0.0002	0.9990 ± 0.0003	0.9989 ± 0.0000	0.9991 ± 0.0002	0.9989 ± 0.0003

The different superscript letters in the same row represent significant differences (*p* < 0.05).

**Table 4:** The population of different bacterial groups in different intestinal sections of quail.

Item	Duodenum	Jejunum	Ileum	Cecum	Colorectum
Total Bacteria	5.98 ± 1.07 ^a^	7.11 ± 0.40 ^bc^	8.83 ± 0.68 ^c^	10.97 ± 1.31 ^d^	11.05 ± 0.64 ^d^
Firmicutes	5.93 ± 0.68 ^a^	7.02 ± 0.75 ^b^	7.93 ± 0.81 ^b^	10.06 ± 1.42 ^c^	10.88 ± 0.94 ^c^
Bacteroidetes	4.76 ± 0.51 ^a^	5.08 ± 0.82 ^ab^	6.45 ± 0.36 ^b^	9.63 ± 0.72 ^c^	9.46 ± 0.39 ^c^
Bacteroides	4.80 ± 0.63 ^a^	5.05 ± 0.42 ^b^	5.87 ± 0.51 ^b^	9.02 ± 0.53 ^c^	8.77 ± 0.45 ^c^

The abundance of bacterial groups was expressed as log10 16S rRNA gene copies/g of fresh luminal contents. The different superscript letters in the same row represent significant difference (*p* < 0.05).

**Table 5:** The concentrations of short-chain fatty acids (SCFAs) in different intestinal sections of quail.

Item	Duodenum	Jejunum	Ileum	Cecum	Colorectum
Acetate	0.0415 ± 0.0361 ^a^	0.0477 ± 0.0243 ^a^	0.0866 ± 0.0764 ^b^	1.3092 ± 0.4760 ^c^	0.7251 ± 0.1653 ^d^
Propionate	0.0303 ± 0.0218 ^a^	0.0287 ± 0.0267 ^a^	0.0401 ± 0.0423 ^a^	0.6788 ± 0.2488 ^b^	0.3898 ± 0.1131 ^c^
Butyrate	—	—	0.0261 ± 0.0236	0.5482 ± 0.1595	0.2348 ± 0.1243
Isobutyrate	—	—	—	0.0511 ± 0.0044	0.0440 ± 0.0074
Valerate	—	—	—	0.0319 ± 0.0251	0.0232 ± 0.0315
Isovalerate	—	—	—	0.0265 ± 0.0353	—

The concentrations of SCFAs were expressed as mg/g of fresh luminal contents. The different superscript letters in the same row represent significant difference (*p* < 0.05). “—” indicates that the SCFA is undetected in the GIT section.

**Table 6:** The abundance of terminal genes for butyrate synthesis in different intestinal sections of quail.

Item	Duodenum	Jejunum	Ileum	Cecum	Colorectum
Butyryl-CoA:acetate-CoA transferase	4.02 ± 0.37 ^a^	3.73 ± 0.50 ^a^	3.99 ± 0.62 ^a^	6.70 ± 0.27 ^b^	6.03 ± 0.44 ^c^
Butyrate kinase	4.13 ± 0.29 ^a^	4.21 ± 0.51 ^a^	5.41 ± 0.59 ^b^	6.82 ± 0.51 ^c^	5.68 ± 0.49 ^d^

The abundance of functional genes was expressed as log10 gene copies of total DNA/g of fresh luminal contents. The different superscript letters in the same row represent significant difference (*p <* 0.05).
